# Chronic Stress in Young German Adults: Who Is Affected? A Prospective Cohort Study

**DOI:** 10.3390/ijerph14111325

**Published:** 2017-10-31

**Authors:** Ronald Herrera, Ursula Berger, Jon Genuneit, Jessica Gerlich, Dennis Nowak, Wolff Schlotz, Christian Vogelberg, Erika von Mutius, Gudrun Weinmayr, Doris Windstetter, Matthias Weigl, Katja Radon

**Affiliations:** 1 Occupational and Environmental Epidemiology and NetTeaching Unit, Institute and Outpatient Clinic for Occupational, Social, and Environmental Medicine, University Hospital Munich (LMU), 80336 Munich, Germany; jessica.gerlich@med.uni-muenchen.de (J.G.); doris.windstetter@gmx.de (D.W.); katja.radon@gmail.com (K.R.); 2 Institute for Medical Informatics, Biometry and Epidemiology-IBE, University of Munich (LMU), 81377 Munich, Germany; berger@ibe.med.uni-muenchen.de; 3 Institute of Epidemiology and Medical Biometry, Ulm University, 89081 Ulm, Germany; jon.genuneit@uni-ulm.de (J.G.); gudrun.weinmayr@uni-ulm.de (G.W.); 4 Institute and Outpatient Clinic for Occupational, Social, and Environmental Medicine Medical Faculty Ludwig-Maximilians-University Munich, Germany and German Center for Lung Research (DZL), 80336 Munich, Germany; dennis.nowak@med.uni-muenchen.de; 5 Max Planck Institute for Empirical Aesthetics, 60322 Frankfurt am Main, Germany; wolff.schlotz@aesthetics.mpg.de; 6 Department of Pediatrics and Adolescent Medicine, University Hospital Carl Gustav Carus Dresden, Technical University Dresden, 01397 Dresden, Germany; christian.vogelberg@uniklinikum-dresden.de; 7 Dr von Hauner Children’s Hospital, LMU Munich Munich, Germany and German Center for Lung Research (DZL), 80336 Munich, Germany; erika.von.mutius@med.uni-muenchen.de; 8 Institute and Outpatient Clinic for Occupational, Social, and Environmental Medicine, University Hospital Munich (LMU), 80336 Munich, Germany; matthias.weigl@med.uni-muenchen.de

**Keywords:** work stress, longitudinal study, psychological effects, generalized estimation equations

## Abstract

We aimed to prospectively assess changes in chronic stress among young adults transitioning from high school to university or working life. A population-based cohort in Munich and Dresden (Germany) was followed from age 16–18 (2002–2003) to age 20–23 (2007–2009) (*n* = 1688). Using the Trier Inventory for the Assessment of Chronic Stress, two dimensions of stress at university or work were assessed: work overload and work discontent. In the multiple ordinal generalized estimating equations, socio-demographics, stress outside the workplace, and job history were additionally considered. At follow-up, 52% of the population were university students. Work overload increased statistically significantly from first to second follow-up, while work discontent remained constant at the population level. Students, compared to employees, reported a larger increase in work overload (adjusted odds ratio (OR): 1.33; 95% confidence interval (95% CI): 1.07, 1.67), while work discontent did not differ between the groups. In conclusion, work overload increases when young adults transition from school to university/job life, with university students experiencing the largest increase.

## 1. Introduction

To promote well-being across an adult’s working life, occupational health specialists face the challenge of determining and preventing stress among employees. Work stress is conceptualized as the interaction of employee characteristics with demands of the personal and work environment [1]. If personal resources of employees are not effective in coping with the demands and pressures of the job, adverse psychological and physiological reactions such as sleeping disorders, cardiovascular effects, chronic pain, and depression may result [2,3,4,5]. Previous research has shown that particularly chronic, rather than acute, stress is associated with adverse health effects [6,7,8]. While acute stress refers to situations that occur only once and begin or end abruptly, chronic stress is related to a daily routine in a constant environment in the absence of effective coping mechanisms [9,10]. Aside from job demands, chronic stress at work includes a persistent lack of need fulfillment (e.g., need for appreciation, autonomy, social support, or meaningful tasks) [10,11].

Chronic stress may already develop early in working life [12] and varies across the lifespan. The current economic crisis in Europe generated investment in training of young people to avoid unemployment and improve job perspectives [13]. In these young people, prevalence of chronic stress might be highest, as recently shown in a German national survey [14]. Many of these young adults are still enrolled at the university or, especially in Germany, in so-called dual training systems where school-based training is combined with training on the job. While the latter are covered by Germany’s Occupational Safety Law [15] (which includes stress prevention at the workplace), no such programs are regularly in place at most German universities. At the same time, many university students face a double burden, as they need to work to earn a living in addition to studying. Others need to do mandatory internships to complete their studies. Research has indicated high levels of stress, anxiety, and depression among university students [16,17]. For example, a prospective study among more than 14,000 university students in the UK showed a significant increase in distress in the transition from school to university life [18]. Only few of the existing studies included peers not studying at universities. None of them followed a prospective design [19,20]. The advantage of using such a design is that the difference in personal stress levels before entering university/working life can be controlled for.

In addition, measurement of stress differed across existing studies with most studies using distress as a proxy of stress levels [21]. For a valid comparison of stress levels among students and non-students, it is important to use an instrument applicable to the school, university and working environment alike. In addition, it should include stress factors outside the workplace/university setting like social stress, worries, lack of social recognition, and worrying memories to control for potential sources of non-job-related chronic stress. The Trier Inventory for the Assessment of Chronic Stress (TICS) considers these dimensions and enables the reliable and comprehensive assessment of stress across various domains including training settings such as schools and universities or in unemployment [6,22]. This scale has been successfully implemented in the above-mentioned cross-sectional national survey of German adults [15].

We aimed to prospectively assess the marginal change in chronic stress following a population-based cohort transitioning from high school to university/working life. In addition, our objective was to compare stress levels among university students and their non-student counterparts. Finally, we wanted to examine chronic stress levels by occupational groups.

## 2. Materials and Methods

### 2.1. Population

The study population consisted of participants of the Study of Occupational Allergy Risks (SOLAR) II [23]. In brief, SOLAR II was aimed to investigate the course of respiratory diseases and atopy in symptomatic and symptom free persons from childhood to young adulthood. Additionally, occupational risk factors were identified to investigate associations between occupational factors, stress and the course of respiratory diseases. SOLAR II is the 2nd follow-up of the International Study on Asthma and Allergies in Childhood (ISAAC) II [24]. ISAAC II was the German part of a multicenter international study intended to investigate the prevalence of asthma and allergies, the sample was chosen using schools in Munich and Dresden as sampling units, and participants at age 10 were studied (4th grade, age range: 9–11) in 1995/1996. It included 7498 children. In 2002/2003, SOLAR I (first follow-up) was started. We re-contacted 3785 of the initial ISAAC II participants, of which 3053 adolescents with an average age of 17 (age range: 19–23) agreed to participate. By SOLAR II (the second follow-up), we re-contacted 2904 participants from SOLAR I, and a total of 2051 of 2904 young adults agreed to participate in 2007/2009. During SOLAR I, participants received the questionnaires between August and January 2003. For SOLAR II, fieldwork was distributed between August 2007 and November 2008, and 100 invitation letters per month and per center were sent out.

**Figure 1:**
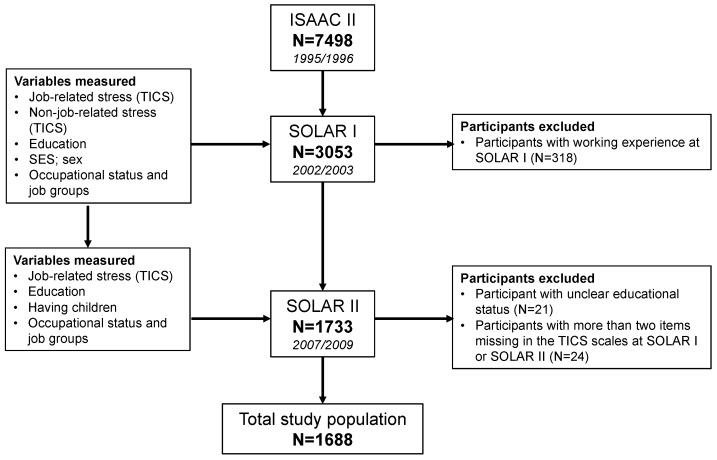
Flow chart of final sample size and the variables measured in each follow-up.

Starting from SOLAR I in 2002–2003, questionnaires included a detailed school and employment history and the TICS version 1.0 [6]. Analyses were restricted to participants who never worked until SOLAR I, excluding 318 subjects with a previous work history (Figure 1). Moreover, 21 participants with unclear educational status and 24 participants with two or more items missing in one of the TICS questionnaires at SOLAR I or SOLAR II were excluded (Figure 1). All participants or their legal guardians provided written informed consent. The Ethical Committees of the Medical Faculty of the University of Dresden, the University of Ulm, and the Ethical Committee of the Bavarian Chamber of Physicians in Munich approved the study (EK 38022007).

### 2.2. Occupational Status and Job Groups

At SOLAR I and SOLAR II, participants reported their current occupational status as well as any jobs they had ever held up until SOLAR I and between SOLAR I and SOLAR II. Occupational status at SOLAR II was categorized as: employee, university student, vocational trainee (dual training system), unemployed, self-employed, and other (i.e., in maternity leave or work disability) [23]. Being employed served as the reference category in the analyses.

Jobs held two years prior to SOLAR II were coded according to the International Standard Classification of Occupations (ISCO-88) by two trained coders [25]. The period of two years was chosen because we considered this amount of time as relevant for the development of chronic stress at SOLAR II. Jobs included regular employment, student jobs, and internships. As subjects could change jobs in the period under study, they were eligible for more than one job category. Using the codes, we constructed five job groups each with at least 90 participants (no vs. yes): clerks (ISCO-88 Major group 4), professionals and technicians (ISCO-88 Major groups 2 and 3), health professions (i.e., with direct patient contact; e.g., medical doctors, physiotherapists, and related associate professionals), plant machine operators (ISCO-88 Major group 8), and elementary occupations (ISCO-88 Major group 9). In each of the job groups, never worked (the no category) in the specific job was used as the reference.

### 2.3. Sociodemographics Covariates

Sex (male vs. female), having children at SOLAR II (no vs. yes), parental socioeconomic status defining high socioeconomic status as at least one parent having ≥12 years of schooling at SOLAR I (high vs. low), highest educational status reported at SOLAR I and SOLAR II (elementary education vs. secondary education, advanced technical and higher education) were included as sociodemographic covariates.

### 2.4. Non-Job-Related Chronic Stress

The Trier Inventory for Chronic Stress (TICS) captures chronic stress considering the following dimensions: work overload, work discontent, social overload, a lack of social recognition, chronic worrying, and stressful memories [22]. They were selected using the systemic requirement-resource model of health [6]. The factorial validity of the TICS was shown by confirmatory analysis in a representative sample [22]. In our analyses, the four TICS sub-scales not directly related to work stress at SOLAR I were included as potential confounders:

- social overload (e.g., “Situations in which I cannot resolve conflicts that I have with others”.);
- lack of social recognition (e.g., “Times where I get little approval for my work”.);
- chronic worrying (e.g., “Times when I worry a lot and cannot stop”.);
- stressful memories (e.g., “Intrusive remembrances of an unpleasant experience”.).

They were asked only once in SOLAR I because they are considered to be persistent over time, especially over a period as short as the five-year period between SOLAR I and SOLAR II (personal communication with Wolfgang Schlotz).

The TICS sub-scales capture the frequency of self-perceived stressful situations in the last 12 months on a five-point Likert scale from “never” (0 points) to “very often” (4 points). A total score in each sub-scale is calculated by summing all item scores; answers are allowed to be missing for up to 2 items and were imputed. Each subscale score was categorized as “low” (≤median), “average” (above median to median +1 standard deviation), and “high” (≥1 standard deviation from the median) frequency of stressful situations [14].

### 2.5. Work-/University-Related Chronic Stress as Outcome

The following job-related TICS sub-scales served as outcomes:

- work discontent (e.g., “Times when I have to perform tasks that I am not at all willing to do”.);
- work overload (e.g., “I have too many tasks to perform”.).

All job-related TICS questions apply to school, university, or job settings alike. As the TICS sub-scales described previously, they were assessed on a five-point Likert scale from “never” to “very often”. Outcomes were categorized like the other TICS domains in “low”, “average”, and “high” using the median and the median +1 standard deviation as cut-off points [14]. Medians and standard deviations of SOLAR I were used for the definition of work discontent and work overload at SOLAR I and SOLAR II.

### 2.6. Statistical Analysis

As the outcomes were measured at two points in time (SOLAR I and SOLAR II), an ordinal generalized estimating equation (GEE) model with an exchangeable correlation structure was used in the analysis. Population average models estimated using GEE describe changes in the population mean based on changes in covariates. They then provide a population-averaged interpretation between exposure(s) and outcome(s) and address the temporal correlation between outcome measures [26,27]. GEEs tell us, for every one unit increase in a covariate across the studied population, how much response would change on average [28]. Given that we had time-varying covariates such as occupational status and level of education, GEE models produce more efficient and unbiased regression parameters than ordinary least squares regression (OLS) [27].

Using R version 3.2.4 (R Foundation for Statistical Computing, Vienna, Austria) [29], we first examined the unadjusted relationships between occupational status, potential confounders, and stress in univariate models. In the next step, we simultaneously included occupational status, sex, parental socioeconomic status, having children, level of education, and time into a mutually adjusted GEE model (Model 1). After that, we additionally controlled for the five job groups (Model 2). Our final model (Model 3) additionally included the four non-work-related TICS dimensions.

Missing values were handled using multiple imputations by chained equations (MICE) [30] assuming that missing values were missing at random. Five imputed data sets were obtained. Combined adjusted odds ratios and their respective confidence intervals were derived using Rubin’s rules [31].

In the sensitivity analyses, we compared multiply imputed data to complete case analyses. We also stratified by sex to check for differences between women and men. In addition, we restricted Model 3 to students.

## 3. Results

### 3.1. Descriptives

The final study population included 1688 participants. Percentage of women was 59% (*n* = 991). About half of the population were still students at SOLAR II, 52% (*n* = 879), whereas 22% (*n* = 376) were employed (Table 1). Considering jobs held during the two years prior to SOLAR II, 19% (*n* = 328) worked as professionals and technicians, 15% (*n* = 260) as plant machine operators, 12% (*n* = 209) in health professions; 10% (*n* = 163) worked as clerks and 6% (*n* = 93) in elementary occupations.

Regarding the outcomes, while median work overload scores did not change considerably between SOLAR I and SOLAR II, the relative frequency of subjects in the high work overload category increased from 17% (*n* = 276) to 21% (*n* = 348). With respect to work discontent, the median score decreased slightly from 13 at SOLAR I to 12 at SOLAR II and relative frequency of participants with high work discontent decreased from 19% (*n* = 316) at SOLAR I to 13% (*n* = 215) at SOLAR II (Table S1).

### 3.2. GEE Models

Estimating population change over the follow-up, work overload increased statistically significantly from SOLAR I to SOLAR II (crude odds ratio (OR): 1.12, 95% confidence interval (95% CI): 1.04, 1.20). Difference became more pronounced when adjusting for other covariates (Table 2 and Table 3).

Compared to employees, students were more likely to report higher levels of work overload at follow-up (fully adjusted Model 3: aOR: 1.33, 95% CI: 1.07, 1.67). Likewise, work overload of self-employed increased significantly compared to the reference group (aOR: 2.55, 95% CI: 1.16, 5.58). Looking at the different job categories, those working at some time in the healthcare sector were more likely to change in a higher work overload category than those never working in this sector (aOR: 1.17, 95% CI: 1.01, 1.37).

Regarding working discontent, it decreased over time (OR: 0.68, 95% CI: 0.63, 0.74) but differences between SOLAR-I and II became none significant after adjustment. In addition, there was no change in work discontent in students compared to employees (aOR: 1.06, 95% CI: 0.84, 1.34), whereas unemployed subjects reported a higher level of work discontent at follow-up than employees (aOR: 2.15, 95% CI: 1.50, 3.09). Of the five job categories studied, working in the healthcare sector (aOR: 0.84, 95% CI: 0.71, 0.99), as a clerk (aOR: 0.83, 95% CI: 0.70, 0.99) or as a plant machine operator (aOR: 0.82, 95% CI: 0.70, 0.96) was inversely associated with work discontent compared to those not working in these sectors (Table 3). Adjustments only marginally changed the results (Table 2 and Table 3).

Looking at the non-job-related chronic stress parameters of Model 3, those in the high chronic worrying category were also more likely to change into a higher work overload category (aOR: 2.89, 95% CI: 2.41, 3.46) and work discontent (aOR: 1.72, 95% CI: 1.45, 2.05) category between SOLAR I and SOLAR II. Social overload was associated with work overload only (aOR: 1.42, 95% CI: 1.20, 1.68). In contrast, lack of social recognition increased the chances of reporting work discontent at follow-up (aOR: 1.94, 95% CI: 1.64, 2.30).

The sensitivity analyses confirmed consistency between multiply imputed data and complete cases analyses (Table S2). After stratification for sex, results remained stable with only minor changes in the odds ratios for men and women (Tables S3 and S4). Restricting the analyses to students, those with part-time jobs reported less work discontent than those not working. In addition, students with part-time jobs in the healthcare sector reported higher work overload compared to students not working in this sector (Table S5).

## 4. Discussion

In this cohort of young adults, we found a substantial increase of work overload from school to working/university life at the population level. Especially affected were university students compared to their employed counterparts. Differences in work overload became even more pronounced for working students. At the same time, university students were more content with what they were doing than at the SOLAR I, which is reflected by less work discontent compared to employees.

Our findings contribute to the current literature on job-related well-being among young adults during their transition into work life. The few existing studies on stress among young workers showed that new psychological work environments and unfamiliar working conditions might lead to an elevated perception of chronic stress and musculoskeletal symptoms among job beginners [32]. Studies among college students indicated that they may become overwhelmed with feelings that there is not enough time to complete all their work adequately. In our study population, this seems particularly true for students who hold part-time jobs attending school at the same time [11,33,34]. Evidence-based interventions for stress reduction should therefore specifically be targeted on the management of work overload among university students [35]. Students with part-time jobs (but not vocational trainees) in the health sector were mostly affected by higher work overload, but also less work discontent compared to full-time students, which is in line with existing literature. However, work discontent perception could change after several years of working in the health sector [36,37].

Work discontent also decreased significantly over time among clerks and machine operators as compared to those not working in such jobs. It might be that young people starting in such jobs can successfully apply their manual skills and capabilities in vocational practice while they were stressed by the intellectual demands at school. For the other occupational groups, we did observe significant associations with chronic job-related stress, which is consistent with previous work by Melchior et al. [5]. Then, participants exposed to high demanding jobs, such as jobs with excessive workload or with extreme high demanding time pressures, had a greater risk of reporting job chronic stress, which in the long term can lead to severe problems such as depression or anxiety disorders. Our findings remained robust after stratifying for gender, which is an indication of lack of effect modification. This is in line with other studies using the same [14,38] or different instruments to assess stress [39].

Overall, our study expands on previous attempts to determine chronic job-related stress among young adults entering university and to compare them to those entering work life. Due to the prospective design, it is possible to discern the temporal sequence between the exposure and outcome. Using the job-history at SOLAR I, we excluded individuals with previous occupational exposure. Our data were collected as part of an extensive protocol that assessed a wide spectrum of health-related information, and participants were not aware of the main hypothesis addressed in the analyses presented in this paper. Therefore, differential misclassification of exposure or outcome is unlikely. Additionally, as almost half of our study population were students, many of the jobs may have been temporary (e.g., student jobs), which had not been investigated previously.

Our study has some limitations. We used a well-established standardized coding instrument (ISCO-88) to define the occupational groups. However, our analyses were restricted to the most prevalent professions in our cohort. Our data relies on the self-report of work-related chronic stress, which may be biased by personality or other reporting bias. Both the type of job selection and chronic stress may be influenced by several individual, social, and cultural characteristics. Accordingly, all estimates were adjusted for a set of a priori defined potential confounders such as sex, socioeconomic status, education, having children, and non-work related chronic stress. Additionally, we did not assess whether the questionnaires were answered during or after the exam period. However, in SOLAR I, participants received the questionnaires between August 2002 and January 2003, which is a period without main exams in Germany. In SOLAR II, questionnaires were sent between August 2007 and November 2008. It might be therefore that some of our students were in an exam or assessment period. Stress measurement in general underlies a random fluctuation, but we do not believe that our results were largely affected by this non-differential misclassification, as our instrument measures chronic stress rather than study load or work load. Data on contextual features such as ethnic background, cultural conception, and social capital were not collected in our study due to legal and logistical reasons. Health status may have conditioned the beginning of professional life, with participants entering the labor market differentially depending on their health status. This so-called healthy hire effect might have caused unmeasured confounding. SOLAR II was based on a population-based cohort. Several methodologies were used to increase participation as far as possible to decrease selection bias. Generalizability of our results should, however, be interpreted carefully, since females and children of parents with higher parental education level were more likely to take part in each follow-up [23].

## 5. Conclusions

In summary, our prospective study of young adults transitioning from school to university and working life indicates that university students were especially affected by work overload, especially those who were concurrently working and studying. This indicates that stress-related interventions might be useful for university students as chronic stress may result in poor health over time. Particularly health professionals reported increased chronic job stress in comparison to other professions, which calls for further measures to reduce psychosocial risks in the healthcare sector.

## Figures and Tables

**Table 1:** Descriptive data of outcomes, exposures, and potential confounders at SOLAR I (2002–2003) and II (2007–2009) in 1688 young Germany adults prior to imputation. Where data for SOLAR I and II remained constant, only data for SOLAR I are given.

	SOLAR I		SOLAR II	
	%	(n)	%	(n)
**Occupation (NA = 5)**				
Employed	0	0	22	376
Student	89	1497	52	879
Apprentice	10	168	19	314
Unemployed	0	0	4	60
Other	1	23	2	42
Self-employed	0	0	1	12
**Clerks**	-	-	10	163
**Professionals and technicians**	-	-	19	328
**Health professions**	-	-	12	209
**Plant machine operators**	-	-	15	260
**Elementary occupations**	-	-	6	93
**Sex**				
Female	59	991	-	-
**Having children (NA = 10)**				
Yes	-	-	3	52
Parental socio economic status ** (NA = 19)				
High	64	1063	-	-
Low	36	606	-	-
**Education (NA = 33)**				
Elementary	74	1220	0	8
Secondary	26	432	26	432
Advanced technical	0	3	12	199
Higher	0	0	62	1043
**Work discontent ^††^ (NA = 17)**				
Low	52	872	68	1137
Average	29	483	19	326
High	19	316	13	215
Median (SD)	13	(3.3)	12	(3.4)
**Work overload ^††^ (NA = 17)**				
Low	55	920	53	882
Average	28	475	27	449
High	17	276	21	348
Median (SD)	20	(5.6)	20	(6.0)
**Social overload (NA = 15)**				
Low	39	659	-	-
Average	43	724	-	-
High	17	290	-	-
Median (SD)	14	(3.4)	-	-
**Lack of social recognition (NA = 11)**				
Low	56	943	-	-
Average	30	499	-	-
High	14	231	-	-
Median (SD)	17	(4.1)	-	-
**Chronic worrying (NA = 17)**				
Low	51	853	-	-
Average	28	474	-	-
High	21	344	-	-
Median (SD)	15	(4.5)	-	-
**Stressful memories (NA = 12)**				
Low	53	882	-	-
Average	26	436	-	-
High	21	358	-	-
Median (SD)	13	(4.6)	-	-

Note: SD: Standard deviation. ^††^: Cut-off points were established based on SOLAR I distribution. ** High: at least one parent having ≥12 years of schooling. NA: Missing Values.

**Table 2:** Unadjusted (OR) and adjusted odds ratios (aOR) after multiple imputation with 95% confidence intervals (95% CI) for work overload using ordinal generalized estimating equation (GEE) models in a prospective cohort of 1688 young adults in Germany.

	Univariate	Model 1 ^#^	Model 2 ^#^	Model 3 ^#^
	OR (95% CI)	aOR (95% CI)	aOR (95% CI)	aOR (95% CI)
**Follow-up**				
SOLAR I	1	-	1	1
SOLAR II	1.12 (1.04, 1.20) *	1.51 (1.23, 1.85) *	1.50 (1.23, 1.84) *	1.55 (1.22, 1.95) *
**Occupation**				
Employed	1	1	1	1
Student	1.01 (0.89, 1.16)	1.30 (1.08, 1.57) *	1.30 (1.08, 1.57) *	1.33 (1.07, 1.67) *
Vocational trainee	0.96 (0.82, 1.13)	1.11 (0.92, 1.33)	1.10 (0.92, 1.33)	1.07 (0.86, 1.34)
Unemployed	0.71 (0.49, 1.03)	0.70 (0.47, 1.05)	0.70 (0.47, 1.05)	0.62 (0.39, 1.00)
Other	1.10 (0.77, 1.57)	1.09 (0.73, 1.62)	1.08 (0.73, 1.61)	1.07 (0.69, 1.67)
Self-employed	2.08 (1.10, 3.94) *	2.32 (1.16, 4.62) *	2.34 (1.16, 4.69) *	2.55 (1.16, 5.58) *
Clerk **				
Yes	1.04 (0.88, 1.24)	-	0.98 (0.82, 1.16)	1.04 (0.87, 1.25)
Professionals and technicians **				
Yes	0.99 (0.87, 1.13)	-	0.95 (0.83, 1.09)	0.94 (0.82, 1.08)
Health professions **				
Yes	1.19 (1.02, 1.38) *	-	1.20 (1.03, 1.41) *	1.17 (1.01, 1.37) *
Plant machine operators **				
Yes	0.81 (0.70, 0.93) *	-	0.91 (0.79, 1.06)	0.92 (0.79, 1.08)
Elementary occupations **				
Yes	1.00 (0.83, 1.22)	-	1.07 (0.87, 1.32)	1.01 (0.81, 1.27)
Sex				
Male	1	1	1	1
Female	1.77 (1.59, 1.97) *	1.78 (1.59, 1.99) *	1.77 (1.58, 1.97) *	1.35 (1.22, 1.53) *
Having children **				
Yes	1.54 (1.19, 1.99) *	1.41 (1.08, 1.83) *	1.42 (1.09, 1.86) *	1.46 (1.10, 1.93) *
**Parental socio economic status**				
High	1	1	1	1
Low	0.93 (0.83, 1.03)	0.93 (0.83, 1.04)	0.94 (0.84, 1.05)	0.88 (0.79, 0.99) *
**Education**				
Elementary	1	1	1	1
Secondary	0.95 (0.86, 1.06)	0.88 (0.76, 1.01)	0.89 (0.77, 1.03)	0.87 (0.75, 1.01)
Advanced technical	1.17 (0.99, 1.39)	0.87 (0.67, 1.13)	0.88 (0.68, 1.14)	0.86 (0.65, 1.15)
Higher	1.08 (0.99, 1.18)	0.74 (0.60, 0.92) *	0.75 (0.60, 0.93) *	0.77 (0.60, 0.98) *
**Social overload ^††^**				
Average	1.50 (1.34, 1.67) *	-	-	1.24 (1.10, 1.41) *
High	2.48 (2.16, 2.85) *	-	-	1.42 (1.20, 1.68) *
**Lack of social recognition ^††^**				
Average	1.46 (1.30, 1.64) *	-	-	1.08 (0.96, 1.23)
High	2.10 (1.82, 2.42) *	-	-	1.16 (0.99, 1.40)
**Chronic worrying ^††^**				
Average	2.26 (1.99, 2.56) *	-	-	1.82 (1.58, 2.09) *
High	4.04 (3.50, 4.66) *	-	-	2.89 (2.41, 3.46) *
**Stressful memories ^††^**				
Average	1.82 (1.61, 2.07) *	-	-	1.20 (1.04, 1.38) *
High	2.59 (2.28, 2.95) *	-	-	1.13 (0.95, 1.35)

Note: ^#^ Each model mutually adjusted for all variables given in the column. * Statistically significant. ** Reference category “No”. **^††^** Reference category “Low”.

**Table 3:** Unadjusted (OR) and adjusted odds ratios (aOR) after multiple imputation with 95% confidence intervals (95% CI) for work discontent using ordinal GEE models in a prospective cohort of 1688 young adults in Germany.

	Univariate	Model 1 ^#^	Model 2 ^#^	Model 3 ^#^
	OR (95% CI)	aOR (95% CI)	aOR (95% CI)	aOR (95% CI)
**Follow-Up**				
SOLAR I	1	1	1	1
SOLAR II	0.68 (0.63, 0.74) *	1.11 (0.90, 1.38)	1.11 (0.90, 1.38)	1.08 (0.86, 1.36)
**Occupation**				
Employed	1	1	1	1
Student	1.25 (1.06, 1.46) *	1.08 (0.87, 1.35)	1.10 (0.88, 1.37)	1.06 (0.84, 1.34)
Apprentice	0.97 (0.80, 1.18)	0.98 (0.80, 1.22)	0.99 (0.80, 1.22)	0.91 (0.72, 1.14)
Unemployed	2.04 (1.48, 2.82) *	2.08 (1.50, 2.88) *	2.10 (1.52, 2.91) *	2.15 (1.50, 3.09) *
Other	1.52 (1.07, 2.18) *	1.49 (1.01, 2.19) *	1.50 (1.02, 2.21) *	1.46 (0.95, 2.26)
Self-employed	0.69 (0.21, 2.20)	0.71 (0.24, 2.14)	0.70 (0.24, 2.05)	0.61 (0.19, 1.98)
Clerk **				
Yes	0.82 (0.69, 0.97) *	-	0.79 (0.66, 0.95) *	0.81 (0.68, 0.98) *
Professionals and technicians **				
Yes	0.91 (0.80, 1.03)	-	0.90 (0.79, 1.02)	0.90 (0.79, 1.04)
Health professions **				
Yes	0.86 (0.73, 1.01)	-	0.84 (0.71, 1.00)	0.80 (0.67, 0.95) *
Plant machine operators **				
Yes	0.84 (0.72, 0.97) *	-	0.81 (0.70, 0.95) *	0.82 (0.70, 0.96) *
Elementary occupations **				
Yes	0.98 (0.79, 1.21)	-	0.99 (0.80, 1.24)	0.94 (0.74, 1.19)
**Sex**				
Men	1	1	1	1
Women	1.11 (1.01, 1.23) *	1.12 (1.01, 1.25) *	1.11 (1.00, 1.23)	0.89 (0.80, 1.00)
Having children **				
Yes	1.10 (0.86, 1.40)	0.91 (0.70, 1.19)	0.89 (0.68, 1.18)	0.89 (0.67, 1.18)
**Parental socioeconomic status**				
High	1	1	1	1
Low	1.00 (0.91, 1.11)	0.96 (0.86, 1.07)	0.95 (0.85, 1.06)	0.91 (0.81, 1.02)
**Education**				
Elementary	1	1	1	1
Secondary	0.75 (0.67, 0.84) *	0.72 (0.62, 0.83) *	0.71 (0.62, 0.82) *	0.70 (0.60, 0.81) *
Advanced technical	0.73 (0.60, 0.88) *	0.64 (0.49, 0.84) *	0.65 (0.49, 0.85) *	0.60 (0.45, 0.81) *
Higher	0.54 (0.48, 0.60) *	0.48 (0.38, 0.61) *	0.48 (0.38, 0.61) *	0.47 (0.37, 0.61) *
**Social overload ^††^**				
Average	1.48 (1.33, 1.64) *	-	-	1.09 (0.96, 1.23)
High	2.02 (1.77, 2.31) *	-	-	1.17 (0.98, 1.38)
**Lack of social recognition ^††^**				
Average	1.60 (1.43, 1.79) *	-	-	1.38 (1.22, 1.57) *
High	2.53 (2.20, 2.89) *	-	-	1.94 (1.64, 2.30) *
**Chronic worrying ^††^**				
Average	1.65 (1.47, 1.86) *	-	-	1.41 (1.23, 1.62) *
High	2.26 (1.99, 2.56) *	-	-	1.72 (1.45, 2.05) *
**Stressful memories ^††^**				
Average	1.53 (1.36, 1.72) *	-	-	1.19 (1.04, 1.36) *
High	1.92 (1.70, 2.16) *	-	-	1.11 (0.94, 1.31)

Note: ^#^ Each model mutually adjusted for all variables given in the column. * Statistically significant. ** Reference category “No”. ^††^ Reference category “Low”.

## Supplementary Materials

The following are available online at www.mdpi.com/1660-4601/14/11/1325/s1, Table S1: Prevalences in each job-related category in SOLAR I and SOLAR II. Rows represent prevalences at baseline (SOLAR I) and columns are prevalences in SOLAR II, Table S2: Adjusted odds ratios for stress outcomes using complete case (ORCC) and multiple imputation (ORMI) with 95% confidence intervals (95% CI) using the Model 3. Associations obtained using ordinal GEE models in a prospective cohort study of 1688 German young adults, Table S3: Adjusted odds ratios after multiple imputation (aOR) and 95% confidence intervals (95% CI) for work discontent and sex using the Model 3, Table S4: Associations obtained using ordinal GEE models in a prospective cohort study of German young adults. Adjusted odds ratios after multiple imputation (aOR) and 95% confidence intervals (95% CI) for work overload and sex using the Model 3, Table S5: Adjusted odds ratios after multiple imputation (aOR) and 95% confidence intervals (95% CI) for job-related chronic stress outcomes using the Model 3 using only the students.
