# Supplementary material for: Chronic Stress in Young German Adults: Who Is Affected? A Prospective Cohort Study
Source: Int J Environ Res Public Health. 2017 Oct 31;14(11):1325. doi: 10.3390/ijerph14111325 (PMC5707964; doi:10.3390/ijerph14111325)
Supplement: Supplementary file 1 [file ijerph-14-01325-s001.pdf]

Table S1: Prevalences in each work-lated categories in SOLAR I and SOLAR II. Rows represent prevalences at baseline (SOLAR I) and columns are prevalences in SOLAR II.

			**SOLAR II**		
			**Work overload**		
**SOLAR I**	**Work overload**		Low	Average	High
		Low	65.5	23.5	11.0
		Average	42.1	33.0	24.9
		High	27.5	25.7	46.7
	**Work discontent**		Low	Average	High
		Low	74.9	16.9	8.2
		Average	66.0	21.3	12.7
		High	51.7	23.5	24.8

Table S2: Adjusted Odds Ratios for stress outcomes using complete cases (OR_{CC}) and multiple imputation (OR_{MI}) with 95% confidence intervals (95%CI) using the Model 3. Associations obtained using ordinal GEE models in a prospective cohort study of German young adults.

		**Work discontent**		**Work overload**	
		$OR_{CC}(95\%CI)$	$OR_{MI}(95\%CI)$	$OR_{CC}(95\%CI)$	$OR_{MI}(95\%CI)$
Occupation	Employed	**Reference**			
	Student	1.02 (0.90;1.15)	1.06 (0.84;1.34)	1.35 (1.20;1.52)	1.33 (1.07;1.67)*
	Apprentice	0.86 (0.76;0.97)	0.91 (0.72;1.14)	1.07 (0.95;1.20)	1.07 (0.86;1.34)
	Unemployed	2.27 (1.86;2.78)	2.15 (1.50;3.09)*	0.66 (0.51;0.85)	0.62 (0.39;1.00)
	Other	1.40 (1.11;1.77)	1.46 (0.95;2.26)	1.04 (0.82;1.31)	1.07 (0.69;1.67)
	Self-employed	0.43 (0.19;1.01)	0.61 (0.19;1.98)	3.71 (2.36;5.83)	2.55 (1.16;5.58)*
Clerk	No	**Reference**			
	Yes	0.78 (0.71;0.86)*	0.81 (0.68;0.98)*	1.04 (0.95;1.15)	1.04 (0.87;1.25)
Professionals and technicians	No	**Reference**			
	Yes	0.90 (0.84;0.97)*	0.90 (0.79;1.04)	0.95 (0.88;1.02)	0.94 (0.82;1.08)
Health professions	No	**Reference**			
	Yes	0.78 (0.71;0.86)*	0.80 (0.67;0.95)*	1.19 (1.10;1.29)*	1.17 (1.01;1.37)*
Plant machine operators	No	**Reference**			
	Yes	0.81 (0.74;0.88)*	0.82 (0.70;0.96)*	0.94 (0.86;1.02)	0.92 (0.79;1.08)
Elementary occupations	No	**Reference**			
	Yes	0.97 (0.85;1.09)	0.94 (0.74;1.19)	1.03 (0.91;1.16)	1.01 (0.81;1.27)
Time	SOLAR I	**Reference**			
	SOLAR II	1.02 (0.91;1.16)	1.08 (0.86;1.36)	1.53 (1.35;1.73)*	1.55 (1.22;1.95)*
Sex	Man	**Reference**			
	Woman	0.90 (0.85;0.96)*	0.89 (0.80;1.00)	1.36 (1.28;1.45)*	1.35 (1.22;1.53)*
Having children	No	**Reference**			
	Yes	0.89 (0.76;1.04)	0.89 (0.67;1.18)	1.50 (1.29;1.74)*	1.46 (1.10;1.93)*
SES	High	**Reference**			
	Low	0.91 (0.86;0.96)*	0.91 (0.81;1.02)	0.88 (0.83;0.94)*	0.88 (0.79;0.99)*
Education	Elementary	**Reference**			
	Secondary	0.69 (0.63;0.75)*	0.70 (0.60;0.81)*	0.88 (0.81;0.95)*	0.87 (0.75;1.01)
	Advanced technical	0.61 (0.52;0.71)*	0.60 (0.45;0.81)*	0.82 (0.71;0.95)*	0.86 (0.65;1.15)
	Higher	0.50 (0.44;0.57)*	0.47 (0.37;0.61)*	0.77 (0.68;0.88)	0.77 (0.60;0.98)*
Social overload	Low	**Reference**			
	Average	1.00 (0.94;1.08)	1.09 (0.96;1.23)	1.25 (1.17;1.34)*	1.24 (1.10;1.41)*
	High	1.11 (1.01;1.22)*	1.17 (0.98;1.38)	1.42 (1.29;1.55)*	1.42 (1.20;1.68)*
Lack of social recognition	Low	**Reference**			
	Average	1.44 (1.35;1.54)*	1.38 (1.22;1.57)*	1.09 (1.02;1.16)*	1.08 (0.96;1.23)
	High	2.15 (1.95;2.36)*	1.94 (1.64;2.30)*	1.17 (1.07;1.29)*	1.16 (0.99;1.40)
Chronic worrying	Low	**Reference**			
	Average	1.43 (1.33;1.54)*	1.41 (1.23;1.62)*	1.78 (1.66;1.92)*	1.82 (1.58;2.09)*
	High	1.70 (1.55;1.86)*	1.72 (1.45;2.05)*	2.86 (2.60;3.15)*	2.89 (2.41;3.46)*
Stressful memories	Low	**Reference**			
	Average	1.17 (1.09;1.26)*	1.19 (1.04;1.36)*	1.21 (1.12;1.30) *	1.20 (1.04;1.38)*
	High	1.12 (1.02;1.22)*	1.11 (0.94;1.31)	1.11 (1.01;1.22)*	1.13 (0.95;1.35)

***: Indicates statistically significance at** 95% **level**

Table S3: Adjusted odds ratios after multiple imputation (aOR) and 95% confidence intervals (95% CI) for **Work discontent** and sex using the Model 3. Associations obtained using ordinal GEE models in a prospective cohort study of German young adults.

		**Both sex** **aOR (95% CI)**	**Women** **aOR (95% CI)**	**Men** **aOR (95% CI)**
Occupation	Employed	1	1	1
	Student	1.06 (0.84;1.34)	1.01 (0.76;1.35)	1.23 (0.79;1.91)
	Apprentice	0.91 (0.72;1.14)	0.89 (0.67;1.19)	0.96 (0.64;1.43)
	Unemployed	2.15 (1.50;3.09)*	2.58 (1.63;4.09)*	1.74 (0.93;3.27)
	Other	1.46 (0.95;2.26)	1.15 (0.67;1.98)	2.45 (1.18;5.09)*
	Self-employed	0.61 (0.19;1.98)	2.01 (0.74;5.47)	NA†
Clerk	No	1	1	1
	Yes	0.81 (0.68;0.98)*	0.77 (0.62;0.96)	0.88 (0.61;1.26)
Professionals and technicians	No	1	1	1
	Yes	0.90 (0.79;1.04)	0.67 (0.53;0.85)*	0.88 (0.71;1.10)
Health professions	No	1	1	1
	Yes	0.80 (0.67;0.95)*	0.67 (0.53;0.85)*	0.97 (0.75;1.25)
Plant machine operators	No	1	1	1
	Yes	0.82 (0.70;0.96)*	0.75 (0.60;0.95)*	0.89 (0.72;1.10)
Elementary occupations	No	1	1	1
	Yes	0.94 (0.74;1.19)	1.08 (0.79;1.48)	0.77 (0.53;1.14)
Time	1	1	1	1
	2	1.08 (0.86;1.36)	1.02 (0.75;1.39)	1.25 (0.87;1.79)
Having children	No	1	1	1
	Yes	0.89 (0.67;1.18)	0.99 (0.72;1.37)	0.69 (0.28;1.71)
SES	High	1	1	1
	Low	0.91 (0.81;1.02)	0.96 (0.83;1.12)	0.80 (0.67;0.97)
Education	Elementary	1	1	1
	Secondary	0.70 (0.60;0.81)*	0.72 (0.58;0.88)*	0.67 (0.53;0.85)*
	Advanced technical	0.60 (0.45;0.81)*	0.63 (0.43;0.92)*	0.54 (0.33;0.89)*
	Higher	0.47 (0.37;0.61)*	0.52 (0.37;0.72)*	0.39 (0.26;0.57)*
Social overload	Low	1	1	1
	Average	1.09 (0.96;1.23)	1.20 (1.02;1.42)*	0.97 (0.81;1.17)
	High	1.17 (0.98;1.38)	1.26 (1.02;1.57)*	1.17 (0.88;1.55)
Lack of social recognition	Low	1	1	1
	Average	1.38 (1.22;1.57)*	1.32 (1.12;1.55)*	1.40 (1.15;1.70)*
	High	1.94 (1.64;2.30)*	1.75 (1.43;2.14)*	2.21 (1.66;2.96)*
Chronic worrying	Low	1	1	1
	Average	1.41 (1.23;1.62)*	1.26 (1.05;1.51)*	1.62 (1.31;2.00)*
	High	1.72 (1.45;2.05)*	1.74 (1.41;2.14)*	1.40 (0.99;1.98)
Stressful memories	Low	1	1	1
	Average	1.19 (1.04;1.36)*	1.23 (1.03;1.48)*	1.18 (0.96;1.46)
	High	1.11 (0.94;1.31)	1.17 (0.94;1.44)	1.09 (0.81;1.47)

*: Indicates statistically significance at 95% level

†: Not avaliable because not enough individuals for estimation

Table S4: Adjusted odds ratios after multiple imputation (aOR) and 95% confidence intervals (95% CI) for **Work overload** and sex using the Model 3.

		**Both sex**<br>**aOR (95% CI)**	**Women**<br>**aOR (95% CI)**	**Men**<br>**aOR (95% CI)**
Occupation	Employed	1	1	1
	Student	1.33 (1.07;1.67)*	1.23 (0.95;1.60)	1.46 (0.94;2.26)
	Apprentice	1.07 (0.86;1.34)	0.92 (0.71;1.20)	1.48 (0.99;2.21)
	Unemployed	0.62 (0.39;1.00)	0.72 (0.41;1.27)	0.44 (0.15;1.24)
	Other	1.07 (0.69;1.67)	0.94 (0.56;1.57)	1.42 (0.60;3.34)
	Self-employed	2.55 (1.16;5.58)*	1.55 (0.55;4.32)	4.41 (1.19;16.32)*
Clerk	No	1	1	1
	Yes	1.04 (0.87;1.25)	1.03 (0.83;1.28)	1.05 (0.73;1.51)
Professionals and techn.	No	1	1	1
	Yes	0.94 (0.82;1.08)	0.86 (0.74;1.01)	1.08 (0.83;1.41)
Health professions	No	1	1	1
	Yes	1.17 (1.01;1.37)*	1.25 (1.02;1.53)*	1.06 (0.83;1.36)
Plant machine operators	No	1	1	1
	Yes	0.92 (0.79;1.08)	0.91 (0.73;1.14)	0.93 (0.74;1.17)
Elementary occup.	No	1	1	1
	Yes	1.01 (0.81;1.27)	0.93 (0.7;1.23)	1.13 (0.78;1.65)
Time	SOLAR I	1	1	1
	SOLAR II	1.55 (1.22;1.95)*	1.48 (1.11;1.96)*	1.50 (0.99;2.28)
Having children	No	1	1	1
	Yes	1.46 (1.10;1.93)*	1.51 (1.10;2.06)*	1.61 (0.81;3.18)
SES	High	1	1	1
	Low	0.88 (0.79;0.99)*	0.86 (0.75;1.00)	0.93 (0.77;1.14)
Education	Elementary	1	1	1
	Secondary	0.87 (0.75;1.01)	0.91 (0.76;1.09)	0.83 (0.63;1.09)
	Advanced technical	0.86 (0.65;1.15)	0.92 (0.64;1.31)	0.89 (0.53;1.49)
	Higher	0.77 (0.60;0.98)*	0.77 (0.57;1.04)	0.88 (0.57;1.38)
Social overload	Low	1	1	1
	Average	1.24 (1.10;1.41)*	1.30 (1.11;1.52)*	1.16 (0.94;1.44)
	High	1.42 (1.20;1.68)*	1.49 (1.22;1.82)*	1.39 (1.02;1.90)*
Lack of social recognition	Low	1	1	1
	Average	1.08 (0.96;1.23)	0.95 (0.81;1.11)	1.36 (1.11;1.66)*
	High	1.16 (0.99;1.40)	1.07 (0.87;1.30)	1.39 (1.01;1.93)*
Chronic worrying	Low	1	1	1
	Average	1.82 (1.58;2.09)*	1.77 (1.48;2.12)*	1.94 (1.55;2.42)*
	High	2.89 (2.41;3.46)*	3.08 (2.46;3.85)*	2.35 (1.67;3.29)*
Stressful memories	Low	1	1	1
	Average	1.20 (1.04;1.38)*	1.09 (0.91;1.31)	1.43 (1.15;1.77)*
	High	1.13 (0.95;1.35)	1.05 (0.85;1.30)	1.34 (0.97;1.84)*

*: Indicates statistically significance at 95% level

Table S5: Adjusted odds ratios after multiple imputation (aOR) and 95% confidence intervals (95%CI) for job-related chronic stress outcomes using the Model 3 using only the students.

		**Work discontent aOR (95%CI)**	**Work overload aOR (95%CI)**
Clerk	No	1	1
	Yes	0.78 (0.62;0.97)*	1.07 (0.87;1.30)
Professionals and technicians	No	1	1
	Yes	0.89 (0.76;1.04)	0.96 (0.81;1.14)
Health professions	No	1	1
	Yes	0.81 (0.67;0.98)*	1.22 (1.02;1.47)*
Plant machine operators	No	1	1
	Yes	0.79 (0.65;0.96)*	0.93 (0.77;1.13)
Elementary occupations	No	1	1
	Yes	0.91 (0.68;1.20)	0.93 (0.72;1.20)
Follow-up	SOLAR I	1	1
	SOLAR II	0.73 (0.34;1.57)	1.53 (0.85;2.76)
Sex	Man	1	1
	Woman	0.82 (0.72;0.95)*	1.35 (1.18;1.56)*
Having children	No	1	1
	Yes	0.88 (0.59;1.30)	1.43 (0.96;2.14)
SES	High	1	1
	Low	0.96 (0.83;1.10)	0.85 (0.74;0.98)*
Education	Elementary	1	1
	Secondary	0.69 (0.58;0.83)*	0.86 (0.72;1.02)
	Advanced technical	0.75 (0.34;1.67)	0.98 (0.52;1.82)
	Higher	0.67 (0.31;1.45)	0.81 (0.44;1.47)
Social overload	Low	1	1
	Average	1.09 (0.94;1.27)	1.31 (1.12;1.52)*
	High	1.25 (1.01;1.54)	1.55 (1.27;1.90)*
Lack of social recognition	Low	1	1
	Average	1.39 (1.19;1.61)*	1.05 (0.91;1.22)
	High	2.05 (1.67;2.52)*	1.13 (0.91;1.38)
Chronic worrying	Low	1	1
	Average	1.49 (1.26;1.77)*	1.98 (1.67;2.35)*
	High	1.95 (1.58;2.42)*	3.64 (2.94;4.52)*
Stressful memories	Low	1	1
	Average	1.22 (1.03;1.44)*	1.25 (1.06;1.48)*
	High	1.07 (0.87;1.33)	1.07 (0.86;1.32)

*: Indicates statistically significance at 95% level

**: No participants with elementary education
